# Development and Validation of the Single Item Narcissism Scale (SINS)

**DOI:** 10.1371/journal.pone.0103469

**Published:** 2014-08-05

**Authors:** Sara Konrath, Brian P. Meier, Brad J. Bushman

**Affiliations:** 1 Lilly Family School of Philanthropy, Indiana University-Purdue University, Indianapolis, Indiana, United States of America; 2 Institute for Social Research, University of Michigan, Ann Arbor, Michigan, United States of America; 3 Department of Psychiatry, University of Rochester Medical School, Rochester, New York, United States of America; 4 Department of Psychology, Gettysburg College, Gettysburg, Pennsylvania, United States of America; 5 School of Communication & Department of Psychology, The Ohio State University, Columbus, Ohio, United States of America; 6 VU University, Amsterdam, the Netherlands; Tilburg University, Netherlands

## Abstract

**Main Objectives:**

The narcissistic personality is characterized by grandiosity, entitlement, and low empathy. This paper describes the development and validation of the Single Item Narcissism Scale (SINS). Although the use of longer instruments is superior in most circumstances, we recommend the SINS in some circumstances (e.g. under serious time constraints, online studies).

**Methods:**

In 11 independent studies (total N = 2,250), we demonstrate the SINS' psychometric properties.

**Results:**

The SINS is significantly correlated with longer narcissism scales, but uncorrelated with self-esteem. It also has high test-retest reliability. We validate the SINS in a variety of samples (e.g., undergraduates, nationally representative adults), intrapersonal correlates (e.g., positive affect, depression), and interpersonal correlates (e.g., aggression, relationship quality, prosocial behavior). The SINS taps into the more fragile and less desirable components of narcissism.

**Significance:**

The SINS can be a useful tool for researchers, especially when it is important to measure narcissism with constraints preventing the use of longer measures.

## Introduction

Some individuals think they are great and special people who should be admired and respected by others. Such people are often called “narcissists.” The term narcissism comes from the mythical Greek character Narcissus, who fell in love with his own image reflected in the water. In the extreme, narcissism can be a clinical disorder [1], however, it is also widely studied as a personality trait in non-clinical populations [2]. The narcissistic personality is characterized by inflated views of the self, grandiosity, self-focus, vanity, and self-importance [3]. Narcissistic individuals have an exceptionally positive view of themselves, and the narcissistic personality is associated with a complex configuration of intrapersonal and interpersonal outcomes [4]. As outlined below, there are many scientific puzzles in the area of narcissism research and a single-item measure of narcissism would give scholars a practical tool that could be used to obtain a better understanding of this trait.

On the one hand, narcissism is associated with some positive intrapersonal outcomes. For example, people scoring higher in narcissism are high in creativity [5], happiness [6], and self-esteem [7], [8], and low in anxiety [9], [10] and depression [10], [11].

On the other hand, narcissism is associated with many negative outcomes such as being prone to defensive and self-protective strategies. When narcissistic people are faced with threats to their self-worth, concepts of worthlessness are immediately activated, and then quickly suppressed [12]. In addition, after receiving negative evaluations they are likely to see problems with the evaluation technique or the evaluator rather than reflect on how to improve [13]. Narcissistic people also have difficulty maintaining healthy interpersonal relationships [14], [15], perhaps because of their relatively low empathy [16], [17] and low commitment to relationship partners [18]. Narcissists believe they are entitled to the admiration and respect of others, and, when they do not get it, they become angry and aggressive [19], [20], [21], [22].

Scholars have tried to reconcile these striking disparities by trying to understand the underlying dynamics of narcissistic cognition, affect, and motivation, within the context of their social interactions [4]. They argue that to fully understand narcissism, we must understand both the grandiose (or overt) and the vulnerable (or covert) aspects of it, and how these change depending on others' approval or disapproval.

Some scholars see the grandiose and vulnerable aspects as existing simultaneously within single individuals. They see narcissistic people as experiencing ongoing vacillations of extremes of self-worth that are dependent upon situations (e.g. success versus failure) and others' evaluations [4], [23]. Other scholars conceptualize two distinct types of narcissism, with different people leaning toward more grandiose (overt) versus more vulnerable (covert) types. Vulnerable and grandiose narcissism both involve feelings of grandiosity, high self-preoccupation, and a strong need for admiration, but vulnerable narcissists appear to be more shy and fragile, and often experience shame and worry that others might negatively evaluate them for their self-focus (see [24], or a review).

Some scholars argue that linking grandiose narcissism with overt qualities and vulnerable narcissism with covert qualities is erroneous, and that grandiose and vulnerable subtypes can both express themselves in overt and covert ways – yet these arguments seem to apply specifically to clinical populations [25]. Regardless of how these aspects of narcissism are specifically defined, the distinction between grandiosity and vulnerability is important because they measure more obvious versus less obvious ways of being narcissistic, respectively.

### Measurement of Narcissism

Personality psychologists have long been interested in measuring narcissism, and have used a wide variety of methods to do so. For example, some scholars have relied on projective techniques such as the Thematic Apperception Test [26], [27] or the Rorschach [27], [28], in which narcissistic themes are extracted from responses to pictures. Other scholars have attempted to use linguistic clues to document narcissistic tendencies (e.g. first person singular pronoun usage; [29], [30]). Still others have used observer-rated Q-sort procedures to assess narcissism [31], [32], or interview-based assessments such as the Diagnostic Interview for Narcissism [33].

Yet the most common way of assessing narcissism (by far) is to use standardized self-report measures. The most widely used measure of the narcissistic personality is the Narcissistic Personality Inventory [34], which measures *grandiose* or *overt* aspects of narcissism. It contains 40 forced-choice items (e.g. “If I ruled the world it would be a better place” versus “The thought of ruling the world frightens the hell out of me”). The NPI can be broken down into a number of subscales (e.g. 7 subscales: [34]; 4 subscales: [35]; 3 subscales: [36]), with 7 subscales being the most commonly used breakdown. The internal reliability of the full scale is .83, with the 7 subscale reliabilities ranging from .50 to .73 [34]. The full scale also has high test-retest reliability after 13 weeks (*r* = .81); the test-retest reliability on the individual subscales is lower (range: .57 to .80; [37]). Other less established measures of narcissism include the Hypersensitive Narcissism Scale (HSNS), which has 10 items and is designed to measure *vulnerable* or *covert* narcissistic tendencies [38], the Five-Factor Narcissism Inventory (FFNI), which has 148 items [39], and the Pathological Narcissism Inventory (PNI), which has 52 items [40]. The latter two scales measure both grandiose (overt) and vulnerable (covert) aspects of narcissism.

Because the NPI and other measures of overt narcissism are quite long, researchers developed the NPI-16 by selecting 16 of the most face valid items across the several domains of the NPI-40 [41]. It is highly correlated with the NPI-40 (*r* = .90) and is internally reliable (α = .72), with a high test-rest reliability (*r* = .85) after five weeks. In addition, it predicts similar personality traits and dependent measures as the longer NPI-40. The major difference, besides length, is that the NPI-16 is unidimensional, whereas the NPI-40 has several subscales. Another short narcissism scale (4 items) was recently developed as part of a longer scale (12 items total) designed to assess three negative interpersonal traits called the “dark triad of personality”—narcissism, psychopathy, and Machiavellianism [42]. This scale was also correlated with the NPI-40 (*r* = .46), with high internal reliability (α's.78-.85), and high test-retest reliability after three weeks (*r* = .87).

In the current paper we develop and validate a single-item measure of narcissism. Single item measures suffer from a number of shortcomings [43], [44]. For example, they are susceptible to random errors of measurement, such as someone accidentally selecting the wrong option on scale point. With multiple items, mistakes like these can average out. Moreover, single item scales can unnaturally simplify multidimensional or complex topics by reducing them to a single question. They can also miss fine-grained distinctions between people by reducing the number of points of precision. For example, a single item might allow 5 different response options, which places individuals into one of five groups. But with 10 items, now responses can range from 1 to 50, which can greatly increase the ability to make fine distinctions between different degrees of a trait.

Yet, when thinking about the desirability of using short personality scales, practical considerations are very important. Time is a precious commodity, and sometimes researchers want to measure several important constructs but only have a limited amount of time. For example, researchers engaging in large nationally representative surveys and field studies are often pressed for time and resources, and including a single item measure could lower this burden. If one scale item would take 20 seconds to read and complete, a 40-item scale would take 13.3 minutes, a 16-item scale would take 5.3 minutes, and a 4-item scale would take 1.3 minutes to complete. While these differences may seem small, this would depend upon the needs of the researcher and the overall burden to the participants.

In addition, in certain circumstances (e.g. online studies), participants have limited attention spans or time available and single item measures can be useful. Including full measures might be psychometrically more valid, but increased participant fatigue may cause errors, low motivation, high dropout, and poor response quality [45].

Another useful situation could be when a measure needs to be assessed across several different time points (e.g. diary studies; experience sampling studies). It can be burdensome to give participants full versions of scales in these cases. Single item scales can also be useful in research settings where people need to pre-test for higher or lower scorers. Finally, such scales are especially useful for pilot testing of new theories, research questions, or methods. In short, whenever there are time or participant constraints, short measures can and should be used, as long as they have adequate psychometric properties and demonstrated validity.

Because of these practical advantages, single item scales have been widely used in prior research to assess a number of constructs. For example, single item measures have been validated for use in the place of frequently used scales like the Self-Esteem Scale [46]: the Single item Self-Esteem Scale – [47], the state form of the State-Trait Anxiety Inventory [48]: one-item state anxiety measure – [49], and the Need to Belong Scale [50]: single-item need to belong measure – [51]. These scales have adequate properties and demonstrated validity. Single item measures have limitations, but there are several situations in which such measures would be so expeditious that their benefits might outweigh their limitations. It is important that researchers carefully consider whether using such measures is appropriate in their studies.

### Overview and Scale Development

In this paper we develop and validate a single item measure of narcissism. We sought to create a measure that would tap into both grandiose and vulnerable aspects of the (non-clinical) narcissistic personality within a single item. The measure is such that in just a few seconds, researchers will be able to obtain a valid measure of a narcissism that is correlated with longer narcissism scales. Across 11 studies, using several different participant populations and dependent measures, we present evidence for the Single Item Narcissism Scale's (SINS) discriminant validity, convergent validity, predictive validity, and test-retest reliability. We further divided the convergent and predictive validity outcomes into ones that are more intrapersonal (i.e. having implications for the self) versus interpersonal (i.e. having implications for others). This will help researchers to quickly determine whether this scale is relevant for their interests.

We chose the wording of the SINS carefully, aiming to create a face valid and easily understood measure of narcissism. In creating this scale, we hoped to capture some less desirable aspects of narcissism while maintaining its ability to predict specific outcomes. In writing this question we used other single-item measures as models [47], [49], [51].

After some pilot testing, the SINS was worded as follows: “To what extent do you agree with this statement: I am a narcissist. (Note: The word ‘narcissist’ means egotistical, self-focused, and vain.).” (See Appendix S1 for final scale.) In pilot testing the item wording originally did not include a definition of narcissism but we found that including one increased the correlation between the SINS and the NPI. Scale responses initially varied from 1 = not very true of me, to 11 = very true of me. We initially chose these endpoints because they are the same ones used for the Single Item Self-Esteem Scale [47]). In later studies we used reduced end points (7 or 5 point scales) to determine which end points were optimal for the scale. See Table 1 for percentage of participants who endorsed each point on the scale.

**Table 1:** Frequencies of each response choice across all studies.

SINS scale point	Study 1	Study 2	Study 3 Time 1	Study 3 Time 2	Study 4	Study 5	Study 6	Study 7	Study 8	Study 9 Time 1	Study 9 Time 2	Study 10	Study 11
*Pop.*	CS	GA	CS	CS	CS	CS	GA	CS	CS	GA	GA	GA	GA
*N*	110	122	141	141	97	107	272	40^1^	133	822	335	206	200
1	8.2%	37.7%	5.7%	9.9%	10.3%	3.4%	32.0%	15.8%	17.9%	47.2%	47.5%	36.0%	50.0%
2	11.8%	14.8%	17.1%	12.1%	21.6%	11.2%	11.4%	31.6%	28.4%	21.5%	20.4%	20.2%	32.5%
3	20.9%	8.2%	18.6%	20.6%	28.9%	21.6%	12.5%	13.2%	17.2%	10.2%	11.4%	13.3%	9.5%
4	14.5%	9.0%	12.9%	14.2%	13.4%	25.9%	5.9%	13.2%	15.7%	9.9%	7.9%	13.3%	7.0%
5	5.5%	7.4%	8.6%	2.8%	12.4%	19.0%	3.3%	21.1%	14.2%	5.6%	6.1%	12.8%	1.0%
6	11.8%	6.6%	6.4%	7.8%	6.2%	7.8%	8.8%	2.6%	5.2%	2.2%	2.9%	2.5%	—
7	10.9%	4.9%	11.4%	14.2%	2.1%	9.5%	10.3%	2.6%	1.5%	3.4%	3.8%	2.0%	—
8	10.9%	4.9%	12.1%	16.3%	4.1%	1.7%	7.7%	—	—	—	—	—	—
9	3.6%	4.1%	6.4%	0.7%	1.0%	0.0%	3.3%	—	—	—	—	—	—
10	0.9%	0.8%	0.0%	0.7%	0.0%	0.0%	1.5%	—	—	—	—	—	—
11	0.9%	1.6%	0.7%	0.7%	0.0%	0.0%	3.3%	—	—	—	—	—	—
Mean	4.66	3.41	4.71	4.64	3.49	4.16	4.00	3.11	3.01	2.25	2.29	2.62	1.77
SD	2.44	2.73	2.49	2.49	1.83	1.61	3.00	1.62	1.57	1.62	1.67	1.64	0.96
Skewness	.36	.98	.35	.24	.92	.30	.65	.46	.49	1.33	1.32	.71	1.25
Kurtosis	−.86	−.10	−1.09	−1.16	.55	−.38	−.81	−.78	−.70	.96	.84	−.54	.96

*Note*: Pop. =  population; N = number of participants; CS =  undergraduates; GA =  general adult.

1 Sample sizes across all but one study were determined so that we had 95% power to detect significant effect sizes of *r* = .20. However for Study 7 we were unable to recruit more participants, meaning that the critical value for significant correlations was higher (*r* = .31) in this study.

We examined readability statistics of the at the following website: http://www.readability-score.com. The SINS has a Flesch Reading Ease score of 64.2 (NPI-16 = 67.2; NPI-40 = 77.9). In this index, higher numbers indicate easier readability. Scores between 60–70 are understood by 13 to 15 year old students (e.g. *Reader's Digest* has a readability index of 65).The Flesch-Kincaid Grade Level is 7.3, confirming that the SINS is readable by people at a 7^th^ grade educational level (NPI-16  =  grade 8; NPI-40  =  grade 4.5). Thus, even though not every respondent is likely to fully understand the rich connotations of the term “narcissist,” the readability data and our inclusion of a definition suggests that typical respondents will be able to understand the meaning of this term.

## Method

All studies were run with the approval of the University of Michigan Institutional Review Board or the Gettysburg College Institutional Review Board. Informed consent was documented in writing for all in-person studies, but for online studies the IRBs waived the requirement to obtain written informed consent. Instead, participants in these studies indicated their consent by selecting a button that said they agreed to participate in the study. All participants were 18 years of age or older. Deidentified datasets are available upon request to researchers who have obtained IRB approval to conduct secondary analyses on them, since participants in our studies did not consent to publicly posting their data.

## Study 1

In Study 1 we provide initial evidence for the validity of the Single Item Narcissism Scale (SINS). In addition to completing the SINS, participants completed another measure of narcissism, and measures of mood, social desirability, individualism-collectivism, and right-wing authoritarianism. Right-wing authoritarianism is a personality trait strongly associated with a conservative political ideology, but it extends beyond beliefs about specific political topics, and is associated with tendencies to follow and obey authority figures, to conform to social norms, and to aggress against people who violate conventional standards of behavior [52].

We expected that the two measures of narcissism would be correlated with each other, and that narcissism would be related to more positive, yet also angrier, moods. In addition we expected either a null [53] or negative [16] relationship between narcissism and social desirability, based on past research. Other research has found that narcissism is negatively related to independent self-construal (individualism), and positively related to interdependence (collectivism; [54]), an effect that we expected to replicate in Study 1. We did not expect the SINS to correlate with right-wing authoritarianism (RWA) because there is no empirical evidence or necessary logical connection that links egotism and political ideology: people on both extremes of the ideological spectrum could theoretically be narcissistic. Thus we include the RWA measure to demonstrate discriminant validity.

### Participants

Participants were originally 111 undergraduates from the University of Michigan. One participant was dropped because he did not complete the SINS, thus, the final sample consisted of 110 participants (40% male; *M*_age_ = 19.7, *SD* = 1.5; 71% Caucasians).

### Procedure

Participants completed a battery of questionnaires consisting of the Single Item Narcissism Scale, the 40-item Narcissistic Personality Inventory [34], the 10-item Marlowe-Crowne Social Desirability Scale [55], the 32-item Individualism-Collectivism Scale [56], and the 20-item PANAS [57], a measure of positive and negative affect. In addition, we included the 20-item Right Wing Authoritarianism Scale [58] to establish the discriminant validity of the SINS. Study 1 was a secondary analysis of an existing dataset that included other unrelated measures (e.g. health behaviors).

### Results and Discussion

#### Relation Between SINS and Demographic Variables

The mean score on the SINS was 4.66 (*SD* = 2.44). There were no gender differences on the SINS, *F*(1,108) = .88, *p* = .35, or the NPI, *F*(1,109) = .33, *p* = .57.

#### Relationship Between SINS and the NPI

The SINS and the NPI were positively correlated (*r* = .40, *p*<.001). The SINS was also positively related to each of the seven NPI subscales: Vanity (*r* = .36, *p*<.001), Exhibitionism (*r* = .34, *p*<.001), Exploitativeness (*r* = .31, *p* = .001), Authority (*r* = .29, *p* = .003), Superiority (*r* = .23, *p* = .02), and Entitlement (*r* = .22, *p* = .02), however, the relationship between the SINS and Self-Sufficiency was not significant, (*r* = .12, *p* = .21). Thus, SINS is a unitary measure that captures several important aspects of grandiose narcissism.

#### Relationship Between the SINS and Other Variables

We next examined the relationship between the SINS, the NPI, and the other measures. Consistent with some past research, in this study social desirability was not related to the NPI (*r* = −.04, *p* = .69), and was negatively related to the SINS (*r* = −.23, *p* = .02). Appearing morally good to others is not a primary concern for those scoring high in narcissism.

As expected based on prior work [54], both the NPI (*r* = −.29, *p* = .002) and the SINS (*r* = −.22, *p* = .02) were negatively related to collectivism, and were positively related to individualism (NPI: *r* = .43, *p*<.001; SINS: *r* = .26, *p* = .006). In addition, neither the NPI (*r* = .04, *p* = .65) nor the SINS (*r* = .11, *p* = .24) were correlated with Right Wing Authoritarianism, which helps to establish the discriminant validity of the SINS.

Both measures of narcissism were similarly related to the various positive affective states, however, they differed in their relationship to negative affective states (see Table 2). Both the SINS and the NPI were positively related to angry states (e.g. irritable, hostile). However, only the SINS was positively related to other negative states (e.g. fear and shame).

**Table 2:** Affective state and narcissism as measured by the SINS and the NPI (Study 1).

Positive Affect	SINS	NPI	Negative Affect	SINS	NPI
Interested	0.11	0.11	Upset	0.16∼	0.12
Excited	0.18∼	0.23*	Irritable	0.34**	0.25**
Inspired	0.21*	0.15	Hostile	0.28**	0.22*
Alert	0.07	0.09	Distressed	0.11	−0.18∼
Active	0.17∼	0.11	Afraid	0.28**	−0.11
Attentive	0.04	0.13	Scared	0.30**	−0.08
Enthusiastic	0.15	0.15	Nervous	0.14	−0.05
Determined	0.24*	0.13	Jittery	0.16∼	−0.02
Proud	0.31**	0.24*	Ashamed	0.26**	−0.09
Strong	0.13	0.31**	Guilty	0.24*	0.06
Overall positive	0.24*	0.24*	Overall negative	0.34***	0.02

Note: ∼p<.10,*p<.05,**p<.01, ***p<.001.

Taken together, Study 1 demonstrates the SINS' construct validity. Not only is the SINS positively related to the NPI, but it replicates the NPI's relationships with social desirability, individualism, collectivism, positive affect, and anger. One major difference that emerges is that the SINS also captures other types of negative affect that are not usually found in grandiose narcissism. This is something that researchers should consider when using this scale, and it is likely related to the fact that with the SINS, participants must directly admit to being narcissistic.

## Study 2

In Study 2, we examined the SINS in a general adult population online. With an online sample we expected a larger age range. Thus, both measures of narcissism should be negatively related to age [59]. We also expected to replicate the finding that narcissism was associated with more individualistic and less collectivistic tendencies [54].

### Participants

Participants were originally 130 adults recruited from Study Response, a paid online psychology research panel administered through Syracuse University. Eight participants were excluded for failing to complete the relevant measures, leaving a final sample of 122 adults (51% male; *M*_age_ = 46.4; *SD* = 12.7; 90% Caucasians).

### Procedure

Participants completed the SINS, the NPI-16 [41], and a different measure of individualism (independent self-construal) and collectivism (interdependent self-construal) — the 24-item Singelis Self-Construal Scale [60]. Study 2 was a secondary analysis of an existing dataset that included other unrelated measures (e.g. political views).

### Results and Discussion

The mean score on the SINS was 3.41 (*SD* = 2.73; See Table 1 for endorsement percentages from all studies). As in Study 1, the SINS, *F*(1,118) = .10, *p* = .76, and the NPI-16, *F*(1,118) = 1.12, *p* = .29, were unrelated to gender. However, both the SINS (*r* = −.17, *p* = .07) and the NPI-16 (*r* = −.16, *p* = .08) had negative marginal correlations with age. These findings are consistent with other research showing that younger adults tend to score higher in narcissism [59].

Consistent with Study 1, the SINS and the NPI-16 were positively correlated (*r* = .45, *p*<.001). However, the relationships with self-construal were more complex. The SINS was positively related to independent self-construal (*r* = .20, *p* = .03), however, in this sample the NPI was not significantly related to independent self-construal (*r* = .14, *p* = 0.14), although the relationship in the predicted direction. The NPI was negatively related to interdependent self-construal, (*r* = −.20, *p* = .03), however, in this sample the SINS was unrelated to interdependent self-construal (*r* = .03, *p* = .78).

Study 2 presents more evidence for the validity and generalizability of the SINS. As expected, both measures of narcissism were negatively related to age. In addition, the SINS was associated with a more independent self-construal, conceptually replicating individualism associations in Study 1. However, only the NPI was associated with a less interdependent self-construal, whereas the SINS was unrelated to interdependence. Past research on narcissism and self-construal has found inconsistent relationships when considering individual studies [54]. Narcissism is sometimes associated with high independence (and not associated with interdependence), and sometimes associated with low interdependence (and not associated with independence). When individual studies are meta-analyzed, the pattern is that narcissism is a combination of high independence and low interdependence [54]. Given this, it is not surprising that we find results that are somewhat inconsistent across Studies 1 and 2. For now, we can conclude that the SINS is positively associated with individualism across two separate measures.

## Study 3

Study 3 examined the test-retest reliability of the SINS in a college student population. We expected that the SINS measured at Time 1 would be highly correlated with itself at Time 2.

### Participants

Participants were 141 undergraduates from the University of Michigan (39% male; *M*_age_ = 19.9; *SD* = 1.1; 70% Caucasian).

### Procedure

Participants completed the SINS online, and then again 10.9 days later in the laboratory. Because the SINS item was embedded within longer questionnaires for an unrelated study, it is unlikely that participants would have remembered their exact SINS score at both time points.

### Results and Discussion

The mean score on the SINS in the online portion of Study 3 was 4.71 (*SD* = 2.49) at Time 1 and 4.64 (*SD* = 2.49) at Time 2 (See Table 1). The SINS was unrelated to gender in this sample (Time 1: *F*(1,138) = .00, *p* = .98; Time 2: *F*(1,139) = .01, *p* = .91). The test-retest correlation was *r* = .79, *p*<.001. In order to examine whether the time between the online and lab portions of the study affected the test-retest reliability, we next split the file into three time segments. If participants recall their original answer and desire to be consistent with it, this recall should be best for those who came into the lab in closer proximity to the time they completed the online survey. In fact, no such pattern emerges: lab visit within one week of completing online survey (*N* = 51), *r* = .72; between one and two weeks (*N* = 51), *r* = .86; over two weeks (*N* = 38), *r* = .77, all *ps*<.001. In addition, when controlling for the number of days between the online and in-lab administrations of the SINS, the correlation was identical, *r* = .79, *p*<.001.

Thus, scores on the SINS appear to be stable, at least over a short period of time (i.e. approximately 11 days). Further evidence for the stability of the SINS comes from a recent research project by our colleagues, who measured the SINS every day for a period of 21 days in a sample of married couples, as part of a larger investigation of intimate partner violence [61]. Upon request, the researchers calculated the internal stability of responses across the 21 day period, and found that internal consistency was extremely high (α = .96; [62]). This confirms Study 3's finding that those who score high on the SINS at one time point are also likely to score high on it at another time point.

## Study 4

Study 4 examines the construct validity of the SINS using different measures. We expected that there would be no relationship or a negative relationship between self-esteem and the SINS, because the SINS measures more undesirable elements of narcissism. In addition, prior research using another very short measure of narcissism (4 items) finds null or negative relationships with measures of self-esteem (See [42], and Table 3).

**Table 3:** Summary of SINS results.

Measure	Correlation with Single-Item Narcissism Scale (current research)	Correlation with 4-item narcissism scale (prior research) [42]
*General properties*		
Other narcissism measures	Study 1, NPI-40: r = .40***	NPI-40: r = .46**
	Study 2, NPI-16: r = .45***	
	Study 7, NPI-16: r = .50**	
	Study 8, NPI-16: r = .48***	
	Study 10, NPI-40: r = .29***	
	Study 10, HSNS: r = .44***	
	Study 10, PNI: r = .41***	
	Study 10, FFNI: r = 43***	
	Study 11, NPI-40: r = .28***	
Test-retest reliability	Study 3: r = .79*** (11 days)	r = .87*** (3 weeks)
	Study 9: r = .78*** (12 days)	
Discriminant validity: Right Wing Authoritarianism	Study 1: r = .11	—
*Demographic variables*		
Gender	Study 1–4, 6: Males = females	Males > females
	Study 7, 9, 10: Males > females	
	Study 5: Gender not reported	
	Study 8: 100% female sample	
Age	Study 1, 3, 4, 5, 7, 8: Not applicable – college student samples	—
	Study 2: r = −.17∼	
	Study 6: r = −.09	
	Study 9: r = −.19***	
	Study 10: r = −.24***	
	Study 11: r = .12∼	
*Intrapersonal outcomes*		
Social desirability	Study 1: r = −.23*	—
	Study 7: r = −.26	
Individualism	Study 1, Triandis: r = .26**	—
(independent self-construal)	Study 2, Singelis: r = .20*	
Collectivism	Study 1, Triandis: r = −.22*	—
(interdependent self-construal)	Study 2, Singelis: r = .03	
Positive affect	Study 1: r = .24*	—
Negative affect	Study 1: r = .34*	—
Self-esteem	Study 4, Rosenberg SE: r = .08	Rosenberg SE: r = −.13*
	Study 5, Rosenberg SE: r = .21*	Single-Item SE: r = −.03
	Study 7, Rosenberg: r = .00	
	Study 8, Rosenberg: r = .05	
	Study 10, Rosenberg: r = −.20**	
Depressive symptoms	Study 4: r = −.16∼	—
Openness	Study 4: r = .02	r = .15*
Conscientiousness	Study 4: r = −.08	r = −.17**
Extraversion	Study 4: r = .20*	r = .15*
Agreeableness	Study 4: r = −.29**	r = −.17**
Neuroticism	Study 4: r = −.02	r = −.10
Risk taking	Study 6: r = .19**	—
Reward preferences	Study 9, non-social rewards: r = .20***	—
	Study 9, social rewards: r = −.26***	
*Interpersonal outcomes*		
Aggressive behavior	Study 5: SINS associated with more aggressive behavior, after a delay	Self-reported aggressive traits
	(i.e. Block 2 but not Block 1 of aggression trials).	Physical aggressiveness: r = .13
		Hostility: r = .44**
		Anger: r = .12
		Verbal aggressiveness: r = .06
Sexual behavior	Study 6:	Short-term mating orientation: r = .34**
	Sexual sensation seeking: r = .16**	Long-term mating orientation: r = .06
	Number of partners in past year: r = .16**	Sexual experience: r = .22**
	SINS more willing to have sex with stranger *	Sociosexual orientation: r = .21**
	SINS more one night stands *	
	SINS less committed relationships **	
Salary entitlement	Study 7: r = .54**	—
Empathy	Study 8, IRI Empathic Concern: r = −.26**	—
	Study 8, IRI Perspective Taking: r = −.19*	
	Study 8, IRI Personal Distress: r = .14∼	
	Study 8: IRI Fantasy: r = −.06	
	Study 10, IRI Empathic Concern: r = −.46***	
	Study 10, IRI Perspective Taking: r = −.26***	
Prosocial behavior	Study 11: SINS associated with less prosocial behavior after an ego threat	—

Note: ∼p<.11, *p<.05, **p<.01, ***p<.001.

We also expected the SINS to correlate positively with extraversion and negatively with agreeableness, as in prior research [35], [42], [63], [64], [65]. Finally, we included a measure of depressive symptoms, which we expected to correlate negatively with narcissism based on prior research [10], [11].

### Participants

Participants were 97 undergraduates from Gettysburg College (46% male; *M*_age_ = 18.9; *SD* = 0.9; 91% Caucasian).

### Procedure

In addition to the SINS, participants completed the Rosenberg Self-Esteem Scale [46], a 50-item Big Five personality trait measure [66], and the Beck Depression Inventory [67].

### Results and Discussion

The mean score on the SINS in Study 4 was 3.49 (*SD* = 1.83; See Table 1). The SINS was unrelated to gender in this sample, *F*(1,95) = .04, *p* = .85. Nor was the SINS related to self-esteem, *r* = .08, *p* = .44. As expected, the SINS was positively correlated with extraversion (*r* = .20, *p* = .05) and negatively correlated with agreeableness (*r* = −.29, *p* = .004). The SINS was unrelated to the other Big 5 traits: neuroticism (*r* = −.02, *p* = .85), openness to experience (*r* = .02, *p* = .83), and conscientiousness (*r* = −.08, *p* = .42). As expected, there was a negative relationship between narcissism and depression (*r* = −.16, *p* = .11), although it was only marginally significant.

The findings from Study 4 indicate that narcissists tend to be outgoing but unpleasant individuals. They also have a slight tendency to score lower in depression.

## Study 5

The purpose of Study 5 was to assess the relationship between narcissism and a behavioral measure of aggression under ego threatening conditions. Based on previous research, we expected an interaction between ego threat and narcissism, with the most aggressive participants being those who scored high in narcissism and who also experienced an ego threat [19], [20], [21], [22].

### Participants

Participants were originally 116 undergraduates from the University of Michigan (age, gender, and ethnicity not recorded). Nine participants were excluded for having incomplete reaction time data. Thus, the final sample consisted of 107 participants.

### Procedure

Participants completed a number of competitive tasks by computer with a partner, who was allegedly in a different room. (In reality, there was no partner). The first task was a very difficult visual task, with random performance feedback given. Participants stated whether lines had arrows on them or not (half the time they did), but the lines and arrows were presented at a subliminal level (17 ms, between two masks). As a manipulation of ego threat, half of the participants were told that the task was a reliable and valid measure of nonverbal intelligence (important task; high ego threat), whereas the other half were told that the task had unknown reliability and validity (unimportant task; low ego threat).

Next, participants completed a competitive reaction time task [68] against a “partner” of the same sex. Within the ethical limits of the laboratory, participants controlled a weapon that could be used to blast their partner with loud, unpleasant noise. The administration of unpleasant noise through headphones is a well-validated measure of laboratory aggression [69].

Participants were told that they and their ostensible partner would have to press a button as fast as possible on each of 25 trials, and that whoever was slower would receive a blast of white noise through a pair of headphones. Participants set the level of noise their partner would receive between 60 dB (*Level 1*) and 105 dB (*Level 10*, about the same volume as a smoke alarm). A no-noise level (*Level 0*) was also provided. They could also control how long their partner suffered by how long they set the noise duration, from 0 seconds to 2.5 seconds. They received randomly determined noise levels and durations from their “partner” during the task.

Finally, participants completed the SINS and the Rosenberg Self-Esteem scale [46] and were debriefed.

### Results and Discussion

The mean score on the SINS in Study 5 was 4.16 (*SD* = 1.61; See Table 1). In this sample, the SINS and self-esteem were positively related (*r* = .21, *p* = .03).

The intensity and duration of the sound blast were each first summed across the 25 trials, then standardized and added together into a single aggression score (Total Aggression). We also calculated an Immediate Aggression (Block 1: first 12 trials) and Delayed/Reactive Aggression (Block 2: last 13 trials) score.

#### Total Aggression

Via stepwise linear regression, we examined the effect of narcissism (mean centered) and task importance (Important versus Unimportant; Step 1), and their interaction (Step 2) on participants' total aggression levels. Based on prior research, we expected that narcissism should be associated with more aggression under ego threatening (important) conditions. For Total Aggression, in Step 1 we found no main effects of narcissism, *β* = .08, *p* = .42, or importance, *β* = .05, *p* = .58, and in Step 2 there was also no interaction, *β* = .07, *p* = .62.

When we conducted a median split on the SINS, the results were slightly different, with an interaction between condition (ego threat versus ego boost) and narcissism (High versus Low). In an ANOVA, we found no main effects of either narcissism or task importance on aggression, *F*s<1. However, we found a significant interaction between narcissism and task importance, *F*(1,103) = 4.23, *p* = .042. Those scoring high in narcissism displayed more Total Aggression when they participated in an ego-threatening important task. However, the use of the full scale points is recommended for data analysis, thus we focus on the results of regression analyses in our interpretations and conclusions.

#### Immediate versus Delayed/Reactive Aggression

In order to compare the two blocks of aggression, a repeated measures ANOVA was conducted, with block (immediate versus delayed) as the within subjects factor and narcissism and importance as between-subjects factors. There were no main effects, all *F*s<1, all *p*s>.40, and of all the possible interactions between these three variables, only the one between block and narcissism emerged as significant, *F*(1,103) = 3.83, *p* = .05 (all other *F*s<1, all *p*s>.60). Narcissism was not significantly related to Immediate Aggression, *β* = −.27, *p* = .14, but it was significantly associated with increased Delayed Aggression, *β* = .35, *p* = .05.

We are not sure why the SINS only predicted delayed or reactive aggressive responses, and because of this, caution is warranted in interpreting these results. Perhaps participants scoring high on the SINS try hard to inhibit aggressiveness at first, but find this difficult after a certain period of time. Future research is needed to better understand the relationship between the SINS and aggressive behavior. Yet there is other evidence from a recent research project from our colleagues that the SINS predicts self-reported intimate partner violence [61]. Married individuals who scored higher on the SINS reported higher aggressive inclinations compared to low scorers. This confirms Study 5's finding that the SINS may at times be associated with aggressiveness, yet this effect may be limited to delayed aggressive behavior, or self-reported measures [61].

## Study 6

Study 6 provides further validation for the SINS by examining the link between narcissism and sexual health behaviors. Past research has found that narcissists take a non-committal approach to sex and relationships, wanting multiple partners and needing low intimacy in their sexual relationships [18], [70]. They are also more prone to risk-taking behavior in general [71]. Thus, we predicted that narcissism, as measured by the SINS, would be positively correlated with risky sexual behaviors (e.g., having multiple sexual partners) and self-reported general risk-taking.

### Participants

Participants were originally 348 adults, but 76 were dropped due to incomplete data. The final sample consisted of 272 adults (43% male; *M*_age_ = 36.1 years; *SD* = 12.8; 86% Caucasian).

### Procedure

Participants completed an online study on dating and sexual behaviors that was posted on Craigslist community discussion boards in large American cities. Participants responded to a number of questions related to their dating and sexual behaviors. For example, they were asked how willing they would be to have sex with an attractive stranger who propositioned them. Participants also completed the 11-item Sexual Sensation Seeking Scale ([72]; α = .86), which is a measure of sexual risk taking (e.g. *I like wild “uninhibited” sexual encounters*), and a more general risk taking scale (5 items; α = .76) that we developed (e.g. *I like to take risks*). Participants were also asked detailed questions about their past sexual experience (e.g. *How many sexual partners have you had in the last 12 months?*).

### Results and Discussion

The mean score on the SINS in Study 6 was 4.00 (*SD* = 3.00; See Table 1). The SINS was unrelated to gender, *F*(1,270) = 2.41, *p* = .12, or age (*r* = −.09, *p* = .16). As expected, the SINS was positively correlated with sexual sensation seeking (*r* = .16, *p* = .009), and general risk taking (*r* = .19, *p* = .002). The SINS was also positively associated with the number of reported sexual and intimate physical partners in the past year (*r* = .16, *p* = .01). Participants who said they would be willing to have sex with an attractive stranger scored higher on the SINS (*M* = 4.48, *SD* = 3.06) than those who were unwilling (*M* = 3.59, *SD* = 2.90), *F*(1,270) = 6.02, *p* = .02, *d* = 0.30. Finally, those who reported engaging in a one night stand at some point in their lives scored higher on the SINS (*M* = 4.30, *SD* = 3.16) than those who did not (*M* = 3.44, *SD* = 2.56), *F*(1,258) = 4.62, *p* = .03, *d* = 0.27.

In addition, participants who were currently in a committed relationship (married, engaged, cohabiting) scored lower on the SINS (*M* = 3.58, *SD* = 2.76) than those who were not (single, separated, or divorced: *M* = 4.43, *SD* = 3.01), or those who were in casual relationships (*M* = 5.48, *SD* = 3.24), *F*(2,249) = 5.77, *p* = .004. This effect remained when controlling for age, *F*(2,243) = 6.88, *p* = .001. Pairwise comparisons found that the committed group differed from the two non-committed groups (*p* = .002 with casual relationships, and *p* = .04 with not involved). The two non-committed groups, however, did not differ from each other (*p* = .12).

Taken together, these findings replicate past work showing that people scoring high in narcissism report engaging in risky sexual behaviors and have difficulty in maintaining long-term committed romantic relationships.

## Study 7

In Studies 7, 8, 9, and 10 we revised the SINS to use only 7 scale points, rather than the original 11, given participants' tendencies to avoid using the higher end of the 11-point scale (See Table 1 for endorsement properties). Although the Single-Item Self-Esteem scale also uses an 11-point scale, people have no problem using the upper end of that scale, perhaps because they are relatively more reluctant to directly admit that they are narcissists.

We also clarify the relationship between the SINS and previously measured variables (i.e. social desirability and self-esteem). Finally, we examine the link between the SINS and salary entitlement [73]. We expected that the SINS would still positively correlate with the NPI, despite the changed scale points. Based on the results of Study 1, we expected a small negative relationship between the SINS and social desirability, and because of the inconsistent results in Studies 4 and 5, we were unsure what to expect in terms of the relationship between the SINS and self-esteem. Study 7 was important in terms of clarifying the nature of this relationship. In addition, we expected a positive relationship between perceptions of deserved salary and both measures of narcissism.

### Participants

Participants were 40 undergraduates from the University of Michigan (58% male; *M*_age_ = 19.8, *SD* = 1.6; 73% Caucasian).

### Procedure

Participants completed the SINS (1 = *not very true of me*, 7 = *very true of me*), the 10-item Marlowe-Crowne Social Desirability Scale [55], the Rosenberg Self-Esteem Scale [46], the NPI-16 [41], and a measure of salary entitlement [73]. For the latter, participants were given a scenario about a fictional company and asked to imagine that they worked there. They were told that the company was in financial trouble and needed to cut salaries. They were given a list of six co-workers and asked to report whether they deserved the same, more, or less than their co-workers (1 = *deserve a much lower salary than co-worker*, 3 = *same*, 5 = *much higher*). The six answers were averaged to create a salary entitlement score (α = .97).

### Results and Discussion

The mean score on the SINS in Study 7 was 3.11 (*SD* = 1.62; See Table 1). The SINS was correlated with the NPI-16, *r* = .50, *p* = .002. Males scored higher (*M* = 3.57, *SD* = 1.69) than females (*M* = 2.53, *SD* = 1.37) on the SINS, *F*(1,36) = 4.20, *p* = .05, but there was no gender difference on the NPI-16, *F*(1,38) = 0.97, *p* = .33. Both the NPI-16, *r* = −.15, *p* = .38, and the SINS, *r* = −.26, *p* = .12, showed non-significant negative correlations with social desirability, but only the NPI-16 was positively associated with self-esteem, *r* = .32, *p* = .05 (SINS: *r* = .00, *p* = .99). As expected, both the NPI-16, *r* = .45, *p* = .005, and the SINS, *r* = .54, *p* = .001, were associated with the belief that one deserved higher salaries than one's coworkers.

## Study 8

In Study 8, we again used the 1–7 endpoint version of the SINS while again examining its relationship with self-esteem to clarify past mixed results (i.e. Studies 4, 5, and 7). In this study we also examine its relationship to participants' dispositional empathy levels. Based on past research demonstrating the connection between narcissism and low empathy [16], we expected that participants who scored higher on the SINS would have lower empathy scores.

### Participants

Participants were 137 undergraduates from the University of Michigan (100% female; age and ethnic background not reported). However, 4 participants did not complete all measures, leaving 133 for analysis.

### Procedure

As part of a larger unrelated study on mental health and well-being, participants completed the SINS (1 = *not very true of me*, 7 = *very true of me*), the NPI-16 [41], the Rosenberg Self-Esteem Scale [46], and a measure of empathy [74]. The Interpersonal Reactivity Index (IRI) is one of the more widely used measures of empathy because of its multidimensional nature. It consists of a 28-item scale with four different 7-item subscales. Empathic Concern measures people's other-oriented feelings of sympathy for the misfortunes of others (e.g. *I often have tender, concerned feelings for people less fortunate than me*). Perspective Taking is a more cognitive or intellectual component, measuring people's tendencies to imagine other people's points of view (e.g. *I sometimes try to understand my friends better by imagining how things look from their perspective*). The Fantasy subscale measures people's tendencies to imaginatively identify with fictional characters in books or movies (e.g. *I really get involved with the feelings of the characters in a novel*). Personal Distress measures more self-oriented feelings of distress during others' misfortunes (e.g. *When I see someone who badly needs help in an emergency, I go to pieces*).

### Results and Discussion

The mean score on the SINS in Study 8 was 3.01 (*SD* = 1.57; See Table 1). The SINS was correlated with the NPI-16, *r* = .48, *p*<.001. Only the NPI was positively associated with self-esteem, *r* = .30, *p* = .001 (SINS: *r* = .05, *p* = .57). The NPI had only one significant relationship with empathy: higher narcissism was associated with lower Perspective Taking, *r* = −.17, *p* = .05. No other correlation was significant (Empathic Concern, *r* = −.13, *p* = .13; Personal Distress, *r* = −.13, *p* = .15; Fantasy, *r* = .03, *p* = .76).

The SINS was better at predicting participants' empathy scores. Those who scored higher on the SINS scored significantly lower on Empathic Concern (*r* = −.26, *p* = .002), and Perspective Taking (*r* = −.19, *p* = .03), and marginally *higher* on the self-oriented Personal Distress scale (*r* = .14, *p* = .097). There was no relationship between the SINS and the Fantasy subscale, *r* = −.06, *p* = .52. The fact that the SINS can distinguish between more other-oriented and self-oriented subscales of the IRI is notable, especially when considering the NPI's comparatively poor predictive validity for a concept so central to the definition of narcissism.

## Study 9

The purpose of Study 9 was to examine the motivational profile of people who score higher versus lower in the SINS. We asked a diverse sample of participants to think about a variety of commonly enjoyed rewards (e.g. favorite food, self-esteem boost) and to rate how much they enjoyed and desired them. Since narcissism is associated with high self-focus and a relatively low interest in others, we expected that people scoring high in the SINS would find social rewards less pleasurable and desirable than non-social ones. Study 9 also examined the test-retest reliability of the SINS in a general adult population, rather than a college student population like Study 3.

### Participants

Participants were a nationally representative sample of 831 American adults from an online respondent panel administered and recruited by Qualtrics, which is an online survey company. They were given a nominal payment for participation. Nine participants were dropped from analyses because they were below the age of 18, leaving a final sample of 822 (31% male; *M*_age_ = 45.0, *SD* = 15.1; 81% Caucasians, 7% African-American, 3% Asian-American, 8% Other or Unidentified). Of these, 335 participants completed the survey a second time approximately 12 days later (*M* = 12.4 days) so we could assess test-retest reliability in a general adult sample.

### Procedure

Participants completed the SINS and also a modified version of the Sensitivity to Reinforcement of Addictive and other Primary Rewards scale [75]. This scale was originally designed to test preferences for addictive drugs, but it was modified for general population studies to include other types of rewards [76], [77]. Participants were asked to think about 2 social rewards (seeing their best friend; doing something that helps others: α = .86) and 4 non-social rewards (eating their favorite food; drinking their favorite alcoholic beverage; receiving a paycheck now or in the past; or receiving a self-esteem boost such as praise: α = .77). Participants rated each reward for how much they liked it and wanted it (1 = *not at all*, 5 = *extremely*). Items were presented in randomized order, and social and non-social reward scores were calculated by averaging all items for each category.

Approximately 12 days later, participants completed the same survey again for an unrelated study. In the current study, we specifically analyzed the test-retest reliability of the SINS.

### Results and Discussion

The mean score on the SINS was 2.25 (*SD* = 1.62) at Time 1 and 2.29 (SD = 1.67) at Time 2 (See Table 1). Males (*M* = 2.71, *SD* = 1.75) scored higher than females (*M* = 2.05, *SD* = 1.51) on the SINS, *F*(1,780) = 28.38, p<.001. In addition, the SINS was negatively related to age, *r* = −.19, *p*<.001.

Social and non-social rewards were simultaneously entered into a regression model to predict participants' SINS scores. Participants who scored higher on the SINS had a higher preference (liking and wanting) for *non-social* rewards, β = .20, *p*<.001, and a lower preference for *social* rewards, β = −.26, *p*<.001. In other words, those scoring higher on the SINS prefer rewards that are more self-related such as eating food, drinking alcohol, receiving self-esteem boosts, and earning money, whereas at the same time they are less likely to prefer social rewards such as seeing their close friends and helping others.

The test-retest correlation for the SINS was *r* = .78, *p*<.001. In order to examine whether the time between the two survey administrations affected the test-retest reliability, we next split the file into three time segments: 1) second survey within one week of completing the first survey (*N* = 24), *r* = .84; 2) between one and two weeks (*N* = 118), *r* = .77; and 3) over two weeks (*N* = 193), *r* = .78, all *ps*<.001. In addition, when controlling for the number of days between the two survey administrations, the correlation was identical, *r* = .78, *p*<.001. Thus, scores on the SINS appear to be stable in a general adult population, at least over a short period of time (i.e. 12 days).

## Study 10

The purpose of Study 10 was to further examine the convergent reliability of the SINS in another nationally representative sample of American adults. In our prior studies we only examined its relationship to the Narcissistic Personality Inventory, but in this study, we also included three other narcissism measures in order to better understand its properties. We expected the SINS to correlate with each of the other narcissism measures. Study 10 also again measured self-esteem and dispositional empathy. As in Study 8, we expected that the SINS would be negatively correlated with empathy. In addition, we included the measure of self-esteem to clarify the association between the SINS and self-esteem, given the mixed findings from our previous studies (see Table 3).

### Participants

Participants were 206 adults recruited from an online respondent panel administered and recruited by the survey company Qualtrics. They were given a nominal payment for participation. The sample was 50.5% male with a mean age of 44.5 (*SD* = 16.3) and with the following ethnic breakdown: 71.4% Caucasians, 11.2% Hispanic-American, 9.7% African-American, 7.3% Asian-American, 0.5% Unidentified, which is similar to U.S. Census Bureau national norms (49% male; 65.1% Caucasian, 15.8% Hispanic-American, 12.3% African-American, 4.5% Asian-American, 2.3% Multiracial or Other; Statistical Abstracts of the United States, 2011).

### Procedure

Participants completed the SINS and three different measures of narcissism in addition to the 40-item Narcissistic Personality Inventory [34].

The Pathological Narcissism Inventory (PNI) is a 52-item measure of more negative aspects of narcissism, rather than “normal” or “healthy” narcissistic tendencies [40]. It consists of seven subscales that assess the higher order factors of *narcissistic grandiosity* (Entitlement Rage, Exploitativeness, Grandiose Fantasy, Self Sacrificing Self Enhancement) and *narcissistic vulnerability* (Contingent Self-Esteem, Hiding the Self, Devaluing Others; see [78]). Narcissistic grandiosity scales are associated with interpersonal problems such as aggression and dominance, while narcissistic vulnerability scales are associated with interpersonal problems related to avoidance and social withdrawal. Overall, the PNI has implications for clinical problems that might occur as a result of high narcissism [40]. Higher numbers indicate higher pathological narcissism.

The Hypersensitive Narcissism Scale (HSNS) is a 10-item measure of *vulnerable* or *covert* narcissistic tendencies [38]. Sample items are “I feel that I have enough on my hands without worrying about other people's troubles” and “When I enter a room I often become self-conscious and feel that the eyes of others are upon me” (1 = *very uncharacteristic or untrue, strongly disagree*; 5 = *very characteristic or true, strongly agree*).

The Five-Factor Narcissism Inventory (FFNI) is a 148-item measure of narcissism as it relates to maladaptive aspects of the five-factor model of personality traits [39]. The FFNI includes 15 subscales that capture both overt (grandiose) and covert (vulnerable) types of narcissism. The subscales are: Reactive Anger, Shame, Indifference, Need for Admiration, Exhibitionism, Authoritativeness, Grandiose Fantasies, Manipulativeness, Exploitativeness, Entitlement, Low Empathy, Arrogance, Acclaim Seeking, Thrill Seeking, and Distrust. For each of these subscales, higher numbers indicate higher narcissism.

Participants also completed the Rosenberg Self-Esteem Scale [46] to again examine the SINS' relationship to self-esteem. They also completed the Empathic Concern and Perspective Taking subscales of the Interpersonal Reactivity Index [74], to again assess both emotional and cognitive empathy, respectively. Including these measures again can help to clarify mixed patterns with respect to self-esteem (Table 3), and can replicate the relationship between the SINS and dispositional empathy.

### Results and Discussion

The mean score on the SINS was 2.62 (*SD* = 1.64; See Table 1). Males (*M* = 2.87, *SD* = 1.70) scored higher than females (*M* = 2.37, *SD* = 1.56) on the SINS, *F*(1,199) = 4.77, p = .03. The SINS was negatively related to age, *r* = −.24, *p*<.001.

The SINS was negatively correlated with self-esteem in this sample, *r* = −.20, *p* = .004. In addition, it was again negatively associated with Empathic Concern, *r* = −.46, *p*<.001, and Perspective Taking, *r* = −.26, *p*<.001. The SINS again had a positive relationship with the NPI-40, *r* = .28, *p*<.001. In Table 4 we present the correlations between the SINS and each narcissism scale, along with its subscales if applicable.

**Table 4:** Correlations between SINS and other measures of narcissism in nationally representative sample (Study 10).

Measure	Type	r
Hypersensitive Narcissism Scale	V	0.44
Narcissistic Personality Inventory (total)	G	0.28
* NPI Entitlement*	G	0.28
* NPI Exhibitionism*	G	0.23
* NPI Exploitativeness*	G	0.23
* NPI Vanity*	G	0.23
* NPI Self Sufficiency*	G	0.20
* NPI Superiority*	G	0.19
* NPI Authority*	G	0.14
Pathological Narcissism Inventory (total)	G & V	0.41
* PNI Devaluing Others*	V	0.43
* PNI Exploitativeness*	G	0.40
* PNI Entitlement Rage*	G	0.37
* PNI Contingent Self-esteem*	V	0.35
* PNI Grandiose Fantasy*	G	0.29
* PNI Hiding Self*	V	0.28
* PNI Self Sacrificial Self Enhancement*	G	0.17
Five-Factor Narcissism Inventory (total)	G & V	0.43
* FF Manipulativeness*	G	0.45
* FF Entitlement*	G	0.44
* FF Reactive Anger*	V	0.43
* FF Arrogance*	G	0.42
* FF Exploitativeness*	G	0.41
* FF Distrust*	V	0.35
* FF Need for Admiration*	V	0.33
* FF Thrill Seeking*	G	0.31
* FF Grandiose Fantasy*	G	0.28
* FF Low Empathy*	G	0.24
* FF Shame*	V	0.22
* FF Authoritative*	G	0.13
* FF Acclaim Seeking*	G	0.08
* FF Exhibitionism*	G	0.08
* FF Indifference*	G	−0.01

Note: Cutoff scores for significance are as follows: *r* = .13, *p*<.10, *r* = .14, *p*<.05, *r* = .18, *p*<.01. G = grandiose narcissism; V = vulnerable narcissism.

We next examined the relationship between the SINS and three other established narcissism measures. First, the SINS showed a significant correlation with the Hypersensitive Narcissism Scale, *r* = .44, *p*<.001, indicating that it is related to both grandiose (e.g. NPI) and vulnerable narcissism. In terms of Pathological Narcissism Inventory, it was related to the PNI overall, *r* = .41, *p*<.001, and to each of its seven subscales (Table 4). This again suggests that the SINS captures both types of narcissism, and especially the more pathological elements of each. Finally, the SINS was related to the FFNI overall, *r* = .43, *p*<.001, and to 12 of the 15 FFNI subscales (all but Indifference, Exhibitionism, and Acclaim Seeking; Table 4).

Overall, Study 10 provides strong and consistent convergent validity for the SINS, and suggests that it may be a good option to use when it is not possible to use these longer scales.

## Study 11

### Participants

Participants were 227 adults who were recruited on MTURK for a small payment. Of these, 27 were excluded for missing data, leaving a final sample of 200 (33% male; *M*_age_ = 36.0, *SD* = 12.4; 86% Caucasian).

### Procedure

After providing informed consent, participants saw what looked like a “captcha” security check asking them to write down three words that were partially hidden in a noisy background image (see Appendix S2). In reality, they were being unobtrusively randomly assigned to ego threatening words (e.g. failure, lose, punish) versus ego boosting words (e.g. success, win, reward). Participants next completed the SINS (1 = *not very true of me*; 5 = *very true of me*) and the NPI-40. We included a 5-point version of the SINS to examine its scale endorsement properties relative to the 7-point version (Table 1).

Participants were then told that the study was over, but that the online survey was created by one of our research students for a research methods class project. We asked them to recommend an overall grade based on their experience taking the study (from A+ to F in third-grade increments, such as B+, B, B−) and also 8 questions that rated the survey on various features (e.g. *The student's online survey was user-friendly*) and the students' suitability for a paid research assistant position (e.g. *Based on the design of the online survey, the student is well-suited for the job*; 1 = *strongly disagree*, 5 = *strongly agree*). All 9 questions were standardized and combined into a single rating scale (α = .86). Participants were told that their responses would be confidential and not shared with the student. Next, we assessed prosocial behavior by asking participants whether they would help the student experimenter by completing one more short study for free (56% agreed to help).

### Results and Discussion

The mean score on the SINS was 1.77 (*SD* = 0.96; Table 1). There were no gender differences on the SINS, *F*(1,197) = .45, *p* = .50, and it was marginally negatively related to age, *r* = −.12, *p* = .11. The SINS and the NPI were positively correlated, *r* = .28, *p*<.001.

#### Ratings of the student

Using regression analysis, we examined the effect of condition (ego threat versus ego boost), narcissism (SINS or NPI), and their interaction on participants' ratings of the student. Condition did not affect the ratings overall, *β* = −.19, *p* = .17, however there was a main effect of the SINS, *β* = −.33, *p* = .001, which was qualified by an interaction with condition, *β* = .32, *p* = .04. In the ego threat condition, higher narcissism (SINS) was associated with lower ratings of the research assistant, *β* = −.32, *p* = .001. In the ego boost condition, there was no association between SINS narcissism and ratings of the student, *β* = −.03, *p* = .75.

We next ran the same analysis with the NPI and found no main effect of condition, *β* = .05, *p* = .49, but a main effect of narcissism such that higher scores were associated with lower student ratings, *β* = −.30, *p* = .003. The interaction was in the right direction, *β* = .18, *p* = .07, but since it was only marginally significant, it is not discussed further. Overall, both narcissism scales were associated with lower ratings of the student, but the SINS was more sensitive to subtle situational cues than the NPI.

#### Helping behavior

Logistic regression analyses were used to examine the effects of condition, narcissism (SINS or NPI), and their interaction on the decision to help (coded 1) or not (coded 0).

For each scale point endorsed on the SINS, there was a 34% lower likelihood of helping behavior, *β* = −.42, *p* = .05, OR = .66 [.427, 1.005]. In addition, being in the ego threat condition was associated with an 88% decline in helping behavior, *β* = −1.50, *p* = .009, OR = .22 [.072, .693]. However, these effects were qualified by a significant interaction, *β* = .70, *p* = .03, OR = 2.01 [1.094, 3.693]. In the ego threat condition, higher narcissism (SINS) scores were associated with a lower probability of helping, *β* = −.42, *p* = .05, OR = 0.66 (0.427, 1.005), whereas there was no association between the SINS and helping in the ego boost condition, *β* = .28, *p* = .21, OR = 1.32 (0.86, 2.03).

For each scale point endorsed on the NPI, there was a 5% lower likelihood of helping, however, this relationship was marginally significant, *β* = −.05, *p* = .095, OR = .95 (.894, 1.009). There was no effect of condition on helping behavior, *β* = −.41, *p* = .17, OR = .67 (.376, 1.182), nor was the interaction significant, *β* = .02, *p* = .71, OR = 1.02 (.934, 1.107).

#### Comparing the 5-point to the 7-point scale

Although the SINS was associated with predictable outcomes even with fewer scale endpoints, as can be seen from Table 1, the use of the 5-point scale makes it even less likely that participants will endorse the higher narcissism options. Thus, we recommend the use of a 7-point scale in future studies.

## Meta-Analysis Comparing SINS to NPI

Using meta-analytic techniques, we examined whether the SINS or the NPI had stronger correlations between some key self-report measures. In order to be included in the meta-analysis, studies had to measure narcissism using both scales (i.e. Studies 1, 2, 7, 8, and 10). The compared measures included individualism and collectivism (Study 1), independent and interdependent self-construal (Study 2), salary entitlement (Study 7), and IRI Empathic Concern and Perspective Taking (Studies 8 and 10).

Because the SINS and NPI data in each study came from the same participants, correlations between the SINS and NPI were used to compute the variance of the difference in correlations between the two scales for each study. This procedure accounts for the fact that SINS and NPI data are not independent and thus their errors are correlated (see [79]).

Mixed-effects meta-analysis for the nine paired correlations revealed that the average correlation between the SINS and the key conceptual variables was .26, with a 95% confidence interval ranging from .16 to .36, whereas the average correlation between the NPI and key conceptual variables was .24, with a 95% confidence interval ranging from .17 to .31. Both confidence intervals exclude the value zero. Importantly, the magnitude of the two correlations did not significantly differ, *r*_difference: SINS-NPI_ = −.025, *Z* = .52, *p* = .32. Thus, the SINS was as strongly correlated with key conceptual variables as the NPI.

A separate mixed-effects meta-analysis was conducted comparing the effect sizes of the correlation between both measures of narcissism and self-esteem (Studies 7, 8, and 10). This analysis revealed that the average correlation between the SINS and self-esteem in Studies 7, 8, and 10 was −.094, with a 95% confidence interval ranging from −.19 to .008, which includes the value zero. In contrast, the average correlation between the NPI and self-esteem in those studies was .23, with a 95% confidence interval ranging from 0.13 to 0.32, which excludes the value zero. Using the same correction for non-independence as reported above (see [79]), we found that the difference in the two narcissism measures' correlations with self-esteem was significant, *r*_difference: SINS-NPI_ = .31, *Z* = 5.47, *p*<.001. Thus, the SINS is not correlated with self-esteem overall, whereas the NPI is significantly and positively correlated with self-esteem.

## General Discussion

Across 11 studies using a wide range of participant samples, we developed a single item measure of narcissism that we recommend for use in certain contexts, such as when time or question quantity is limited (see Table 3 for summary of results). The SINS correlates positively with several narcissism measures, and has similar predictive outcomes as them. Importantly for narcissism researchers, the SINS is related to both grandiose and vulnerable aspects of narcissism, making it desirable when researchers want to assess narcissism as an overall construct, rather than one specific kind of it. Of course, when using the SINS it is impossible to know which specific aspects of narcissism are being assessed. Therefore, researchers who are interested in specifically assessing grandiose *or* vulnerable narcissism should use appropriate measures in their studies.

People scoring higher on the SINS report both positive and negative intrapersonal outcomes. For example, they report more positive affect (Study 1), more extraversion (Study 4), and marginally less depression (Study 4). Yet the SINS is also associated with less desirable intrapersonal outcomes, for example, less agreeableness (Study 4), and more anger, shame, guilt, and fear (Study 1). The SINS is also related to negative interpersonal outcomes, such as delayed/reactive aggression (Study 5), having less committed relationships with others (Study 6), and showing less prosocial behavior when ego threatened (Study 11).

The relationship between the SINS and self-esteem is inconsistent, with the overall finding of a null relationship (see Table 3 and the meta-analysis). This inconsistency might be explained by differences in study populations, but in any case, this indicates that in general, researchers must be aware that people scoring higher on the SINS do not see themselves in overly positive terms, unlike those scoring high in narcissism as measured by the NPI.

The complicated positive and negative intrapersonal portrait of narcissists when measured with the SINS suggests that this scale may capture more fragile, pathological, and unhealthy aspects of narcissism. Not only do these people think they are great, but they also suffer from feelings of shame, guilt, and fear.

### Strengths and Limitations

Among the strengths of this paper is that we conduct 11 independent studies (total N = 2,250), across a variety of participant populations, to thoroughly demonstrate the SINS' psychometric properties. The SINS has convergent validity that is at least as good as other short measures of narcissism, based on its similarly sized correlations with other narcissism measures (See Table 3). For some aspects of validity, we provide more psychometric evidence than other short narcissism scales have provided to date [42]. For example, in terms of criterion validity, the SINS correlates with variables that are centrally related to the narcissism construct such as individualism/collectivism, empathy, and entitlement. In terms of construct validity, the SINS behaves in predictable ways based on previous research in narcissism: we found that it was associated with a number of outcomes, including behavioral ones, such as social desirability, risk taking tendencies, reward preferences, prosocial behavior, and aggressive behavior. Previous short narcissism scales have not reported on such findings.

Yet our studies, like all studies, include some limitations. We recognize that some readers may be skeptical about whether simply asking people if they are narcissistic is an appropriate measure of narcissism, given that narcissism is associated with a host of defensive processes. Are people really aware of their own levels of narcissism? We would argue that, based on the evidence from the current studies, people who are willing to admit that they are relatively more narcissistic than others, actually are. This is in line with prior research finding that high narcissism scorers (on the NPI) were aware that they were more arrogant, condescending, argumentative, critical, and prone to bragging than low scorers [80]. We have simply taken these ideas one step further by directly asking them if they are narcissistic. We note, however, that our scale is more face valid than longer narcissism scales, and therefore, impression management concerns could potentially play a larger role. Indeed, we found that people who score high in social desirability have lower scores on the SINS, suggesting that those who worry about pleasing others are less likely to agree that they are narcissistic. Researchers should consider these issues when making the decision to include the SINS versus longer scales in their studies. In addition, future researchers might test alternate wordings of a single-item narcissism scale to reduce potential negative connotations associated with the word “narcissist.”

Another limitation is that compared to other single item scales (e.g. self-esteem), the correlations between the SINS and its comparable longer scale (e.g. the NPI) are relatively smaller. For example, the correlation between the single-item self esteem scale and the Rosenberg self esteem scale is in the order of .70 to .80 [47]. The relatively smaller correlation between the SINS and the NPI suggest that the SINS is capturing some different aspects of narcissism than other longer measures. Still, it is similar in size to the correlation between another short measure of narcissism and the NPI (see Table 3 and [42]).

Although the SINS does predict theoretically relevant behaviors, because it consists of only a single item it is not as reliable as longer measures [43], [44]. Thus, when statistical power is low or effect-size estimates are expected to be small, a longer and more reliable measure of narcissism is recommended. Researchers who are interested in detecting fine differences in narcissism levels should also use a longer measure.

## Conclusions

A number of longer measures currently exist to assess narcissism, and many of them are have high reliability and validity. Thus, we believe that this single item measure should only be used when it would be difficult or impossible to include a longer narcissism scale. For example, single-item scales can be useful for studies in which every single question counts in terms of time or participant attention levels (e.g. online studies, large nationally representative surveys, field studies in which a single page on a clipboard is an ideal survey length). In addition, this measure might be useful when using interactive electronic data collection techniques such as text messaging, EMA, or smartphone surveys, in which each number or response given takes effort for participants. Yet, in typical laboratory settings, we recommend the use of longer narcissism scales. Future studies will help us better understand the predictive properties of the SINS, but for now, the SINS is one useful tool that can help to assess the complex aspects of narcissism with one single item.

## Supporting Information

Appendix S1 **The Single-Item Narcissism Scale (SINS).** This file includes the full text of the Single-Item Narcissism Scale (SINS).
(DOCX)

Appendix S2 **Study 11 experimental manipulation materials.** This file includes the images that were used to manipulate ego threat versus ego boost in Study 11.
(DOCX)

## References

[1] APA (2000) Diagnostic and statistical manual of mental disorders. 4th edition, TR; Washington, DC.

[2] Campbell WK, Miller JD (2011) The handbook of narcissism and narcissistic personality disorder. New York: Wiley.

[3] Twenge J, Konrath S, Foster J, Campbell WK, Bushman B (2008) Egos inflating over time: a cross-temporal meta-analysis of the Narcissistic Personality Inventory. Journal of Personality 76: 875–902 discussion 903–918. 18507710 10.1111/j.1467-6494.2008.00507.x

[4] Morf CC, Rhodewalt F (2001) Unraveling the Paradoxes of Narcissism: A Dynamic Self-Regulatory Processing Model. Psychological Inquiry 12: 177–196.

[5] Raskin R (1980) Narcissism and creativity: Are they related? Psychological Reports 46: 55–60.

[6] Rose P (2002) The happy and unhappy faces of narcissism. Personality and Individual Differences 33: 379–391.

[7] Campbell WK, Rudich EA, Sedikides C (2002) Narcissism, Self-Esteem, and the Positivity of Self-Views: Two Portraits of Self-Love. Personality and Social Psychology Bulletin 28: 358–368.

[8] Locke KD (2009) Aggression, narcissism, self-esteem, and the attribution of desirable and humanizing traits to self versus others. Journal of Research in Personality 43: 99–102.

[9] Raskin R, Novacek J (1989) An MMPI description of the narcissistic personality. Journal of Personality Assessment 53: 66–80. 2918459 10.1207/s15327752jpa5301_8

[10] Watson P, McKinney J, Hawkins C, Morris RJ (1988) Assertiveness and Narcissism. Psychotherapy: Theory/Research/Practice/Training 25: 125–131.

[11] Sedikides C, Rudich EA, Gregg AP, Kumashiro M, Rusbult C (2004) Are Normal Narcissists Psychologically Healthy?: Self-Esteem Matters. Journal of Personality and Social Psychology 87: 400–416. 15382988 10.1037/0022-3514.87.3.400

[12] Horvath S, Morf CC (2009) Narcissistic defensiveness: Hypervigilance and avoidance of worthlessness. Journal of Experimental Social Psychology 45: 1252–1258.

[13] Kernis MH, Sun C-R (1994) Narcissism and reactions to interpersonal feedback. Journal of Research in Personality 28: 4–13.

[14] Campbell WK (1999) Narcissism and Romantic Attraction. Journal of Personality and Social Psychology 77: 1254–1270.

[15] Campbell WK, Bush CP, Brunell AB, Shelton J (2005) Understanding the Social Costs of Narcissism: The Case of the Tragedy of the Commons. Personality and Social Psychology Bulletin 31: 1358–1368. 16143668 10.1177/0146167205274855

[16] Watson P, Grisham S, Trotter M, Biderman M (1984) Narcissism and empathy: validity evidence for the Narcissistic Personality Inventory. Journal of Personality Assessment 48: 301–305. 16367529 10.1207/s15327752jpa4803_12

[17] Bushman BJ, Bonacci AM, van Dijk M, Baumeister RF (2003) Narcissism, Sexual Refusal, and Aggression: Testing a Narcissistic Reactance Model of Sexual Coercion. Journal of Personality and Social Psychology 84: 1027–1040. 12757146 10.1037/0022-3514.84.5.1027

[18] Campbell WK, Foster C, Finkel EJ (2002) Does Self-Love Lead to Love for Others? A Story of Narcissistic Game Playing. Journal of Personality and Social Psychology 83: 340–354. 12150232 10.1037/0022-3514.83.2.340

[19] Baumeister R, Smart L, Boden JM (1996) Relation of Threatened Egotism to Violence and Aggression: The Dark Side of High Self-Esteem. Psychological Review 103: 5–33. 8650299 10.1037/0033-295x.103.1.5

[20] Baumeister R, Bushman B, Campbell WK (2000) Self-Esteem, Narcissism, and Aggression: Does Violence Result From Low Self-Esteem or From Threatened Egotism? Current Directions in Psychological Science 9: 26–29.

[21] Bushman BJ, Baumeister RF (1998) Threatened Egotism, Narcissism, Self-Esteem, and Direct and Displaced Aggression: Does Self-Love or Self-Hate Lead to Violence? Journal of Personality and Social Psychology 75: 219–229. 9686460 10.1037//0022-3514.75.1.219

[22] Konrath S, Bushman B, Campbell WK (2006) Attenuating the link between threatened egotism and aggression. Psychological Science 17: 995–1001. 17176433 10.1111/j.1467-9280.2006.01818.x

[23] Rhodewalt F, Madrian JC, Cheney S (1998) Narcissism, self-knowledge organization, and emotional reactivity: The effect of daily experiences on self-esteem and affect. Personality and Social Psychology Bulletin 24: 75–87.

[24] Arble EP (2008) Evaluating the psychometric properties of the hypersensitive narcissism scale: Implications for the distinction of covert and overt narcissism. Masters Theses and Doctoral Dissertations: 236.

[25] Pincus AL, Lukowitsky MR (2010) Pathological narcissism and narcissistic personality disorder. Annual Review of Clinical Psychology 6: 421–446. 10.1146/annurev.clinpsy.121208.131215 20001728

[26] Grayden C (1958) The Relationship Between Neurotic Hypochondriasis and Three Personality Variables: Feeling of Being Unloved, Narcissism, and Guilt Feelings: New York University, School of Education.

[27] Harder DW (1979) The assessment of ambitious-narcissistic character style with three projective tests: The early memories, TAT, and Rorschach. Journal of Personality Assessment 43: 23–32. 16367044 10.1207/s15327752jpa4301_3

[28] Urist J (1977) The Rorschach test and the assessment of object relations. Journal of Personality Assessment 41: 3–9. 845776 10.1207/s15327752jpa4101_1

[29] Exner JE (1973) The self focus sentence completion: A study of egocentricity. Journal of Personality Assessment 37: 437–455. 4592140 10.1080/00223891.1973.10119902

[30] Raskin R, Shaw R (1988) Narcissism and the use of personal pronouns. Journal of Personality 56: 393–404. 3404383 10.1111/j.1467-6494.1988.tb00892.x

[31] Wink P (1992) Three Types of Narcissism in Women from College to Mid-Life. Journal of Personality 60: 7–30. 1560332 10.1111/j.1467-6494.1992.tb00263.x

[32] Wink P, Gough HG (1990) New narcissism scales for the California Psychological Inventory and MMPI. Journal of Personality Assessment 54: 446–462. 2348334 10.1080/00223891.1990.9674010

[33] Gunderson JG, Ronningstam E, Bodkin A (1990) The diagnostic interview for narcissistic patients. Archives of general psychiatry 47: 676. 2360861 10.1001/archpsyc.1990.01810190076011

[34] Raskin R, Terry H (1988) A Principal-Components Analysis of the Narcissistic Personality Inventory and Further Evidence of Its Construct Validity. Journal of Personality and Social Psychology 54: 890–902. 3379585 10.1037//0022-3514.54.5.890

[35] Emmons RA (1987) Narcissism: theory and measurement. Journal of Personality and Social Psychology 52: 11. 3820065 10.1037//0022-3514.52.1.11

[36] Kubarych TS, Deary IJ, Austin EJ (2004) The Narcissistic Personality Inventory: factor structure in a non-clinical sample. Personality and Individual Differences 36: 857–872.

[37] del Rosario PM, White RM (2005) The Narcissistic Personality Inventory: Test–retest stability and internal consistency. Personality and Individual Differences 39: 1075–1081.

[38] Hendin HM, Cheek JM (1997) Assessing hypersensitive narcissism: A reexamination of Murray's Narcism Scale. Journal of Research in Personality 31: 588–599.

[39] Glover N, Miller JD, Lynam DR, Crego C, Widiger TA (2012) The Five-Factor Narcissism Inventory: A Five-Factor Measure of Narcissistic Personality Traits. Journal of Personality Assessment 94: 500–512. 22475323 10.1080/00223891.2012.670680

[40] Pincus AL, Ansell EB, Pimentel CA, Cain NM, Wright AG, et al (2009) Initial construction and validation of the Pathological Narcissism Inventory. Psychological Assessment; Psychological Assessment 21: 365. 19719348 10.1037/a0016530

[41] Ames DR, Rose P, Anderson CP (2006) The NPI-16 as a short measure of narcissism. Journal of Research in Personality 40: 440–450.

[42] Jonason PK, Webster GD (2010) The dirty dozen: a concise measure of the dark triad. Psychological Assessment 22: 420. 20528068 10.1037/a0019265

[43] Nunnally JC, Bernstein IH (1994) Psychometric theory. McGraw-Hill, New York.

[44] Spector PE (1992) Summated rating scale construction: An introduction. London: Sage Publications.

[45] Cape P (2010) Questionnaire length, fatigue effects and response quality revisited.

[46] Rosenberg M (1965) Society and the adolescent self-image. Princeton, NJ: Princeton University Press.

[47] Robins RW, Hendin HM, Trzesniewski KH (2001) Measuring Global Self-Esteem: Construct Validation of a Single-Item Measure and the Rosenberg Self-Esteem Scale. Personality and Social Psychology Bulletin 27: 151–161.

[48] Speilberger CD (1983) Manual for the State-Trait Anxiety Inventory. Consulting Psychologists Press. Palo Alto, CA.

[49] Davey HM, Barratt AL, Butow PN, Deeks JJ (2007) A one-item question with a Likert or Visual Analog Scale adequately measured current anxiety. Journal of clinical epidemiology 60: 356–360. 17346609 10.1016/j.jclinepi.2006.07.015

[50] Leary MR, Kelly KM, Cottrell CA, Schreindorfer LS (2007) Individual differences in the need to belong: Mapping the nomological network. Unpublished manuscript, Duke University.

[51] Nichols AL, Webster GD (2013) The single-item need to belong scale. Personality and Individual Differences in press.

[52] Altemeyer B (1981) Right-wing authoritarianism: University of Manitoba Press.

[53] Auerbach JS (1984) Validation of two scales for narcissistic personality disorder. Journal of Personality Assessment 48: 649–653. 6520692 10.1207/s15327752jpa4806_13

[54] Konrath S, Bushman B, Grove T (2009) Seeing my world in a million little pieces: narcissism, self-construal, and cognitive-perceptual style. Journal of Personality 77: 1197–1228. 19558438 10.1111/j.1467-6494.2009.00579.x

[55] Strahan R, Gerbasi KC (1972) Short, homogeneous versions of the Marlow-Crowne Social Desirability Scale. Journal of Clinical Psychology 28: 191–193.

[56] Triandis HC (1995) Individualism and Collectivism. Boulder, CO: Westview Press.

[57] Watson D, Clark LA, Tellegen A (1988) Development and Validation of Brief Measures of Positive and Negative Affect: The PANAS Scales. Journal of Personality and Social Psychology 54: 1063–1070. 3397865 10.1037//0022-3514.54.6.1063

[58] Altemeyer B (1989) Enemies of freedom: Understanding right-wing authoritarianism. San Francisco: Jossey-Bass.

[59] Foster JD, Keith Campbell W, Twenge JM (2003) Individual differences in narcissism: Inflated self-views across the lifespan and around the world. Journal of Research in Personality 37: 469–486.

[60] Singelis TM (1994) The Measurement of Independent and Interdependent Self-Construals. Personality and Social Psychology Bulletin 20: 580–591.

[61] Bushman B, DeWall CN, Pond R, Hanus M (2014) Partner rejection predicts aggressive inclinations among people high in self-esteem and narcissism. Under review.

[62] Pond R (2014) Internal stability of the SINS across a 21-day period. Personal communication: Sent to Brad Bushman via email on 2252014.

[63] Corbitt EM (2002) Narcissism from the perspective of the five-factor model. In: Costa P, Widiger TA, editors. Personality disorders and the five-factor model of personality. Washington, DC: APA.

[64] Raskin R, Hall CS (1981) The Narcissistic Personality Inventory: Alternative form reliability and further evidence of construct validity. Journal of Personality Assessment 45: 159–162. 16370732 10.1207/s15327752jpa4502_10

[65] Trull T, McCrae R (2002) A five-factor perspective on personality disorder research. In: Costa P, Widiger TA, editors. Personality disorders and the five-factor model of personality. Washington, DC: APA.

[66] Goldberg LR (1999) A broad-bandwidth public domain personality inventory measuring the lower-level facets of several five-factor models. In: Mervielde I, Deary I, De Fruyt F, Ostendorf F, editors. Personality Psychology in Europe. Tilburg, the Netherlands: Tilburg University Press.

[67] Beck AT, Steer RA, Carbin MG (1988) Psychometric properties of the Beck Depression Inventory: Twenty-five years of evaluation. Clinical Psychology Review 8: 77–100.

[68] Taylor SP (1967) Aggressive behavior and physiological arousal as a function of provocation and the tendency to inhibit aggression. Journal of Personality 35: 297–310. 6059850 10.1111/j.1467-6494.1967.tb01430.x

[69] Giancola PR, Zeichner A (2006) Construct validity of a competitive reaction-time aggression paradigm. Aggressive Behavior 21: 199–204.

[70] Foster JD, Shrira I, Campbell WK (2006) Theoretical models of narcissism, sexuality, and relationship commitment. Journal of Social and Personal Relationships 23: 367–386.

[71] Campbell WK, Goodie AS, Foster JD (2004) Narcissism, confidence, and risk attitude. Journal of Behavioral Decision Making 17: 297–311.

[72] Kalichman SC, Rompa D (1995) Sexual sensation seeking and sexual compulsivity scales: validity, and predicting HIV risk behavior. Journal of Personality Assessment 65: 586–601. 8609589 10.1207/s15327752jpa6503_16

[73] Campbell WK, Bonacci AM, Shelton J, Exline JJ, Bushman BJ (2004) Psychological Entitlement: Interpersonal Consequences and Validation of a Self-Report Measure. Journal of Personality Assessment 83: 29–45. 15271594 10.1207/s15327752jpa8301_04

[74] Davis M (1983) Measuring individual differences in empathy: Evidence for a multidimensional approach. Journal of Personality and Social Psychology 44: 113–126.

[75] Goldstein R, Woicik P, Moeller S, Telang F, Jayne M, et al (2010) Liking and wanting of drug and non-drug rewards in active cocaine users: the STRAP-R questionnaire. Journal of Psychopharmacology 24: 257–266. 18801822 10.1177/0269881108096982 PMC2820142

[76] Bushman BJ, Moeller SJ, Crocker J (2011) Sweets, Sex, or Self-Esteem? Comparing the Value of Self-Esteem Boosts With Other Pleasant Rewards. Journal of Personality 79: 993–1012. 21950264 10.1111/j.1467-6494.2011.00712.x PMC3137697

[77] Bushman BJ, Moeller SJ, Konrath S, Crocker J (2012) Investigating the Link Between Liking Versus Wanting Self-Esteem and Depression in a Nationally Representative Sample of American Adults. Journal of Personality 80: 1453–1469. 22329486 10.1111/j.1467-6494.2012.00781.x

[78] Wright AG, Lukowitsky MR, Pincus AL, Conroy DE (2010) The higher order factor structure and gender invariance of the Pathological Narcissism Inventory. Assessment 17: 467–483. 20634422 10.1177/1073191110373227

[79] Borenstein M, Hedges LV, Higgins JP, Rothstein HR (2011) Introduction to meta-analysis: John Wiley & Sons.

[80] Carlson EN (2012) Honestly Arrogant or Simply Misunderstood? Narcissists' Awareness of their Narcissism. Self and Identity 12: 259–277.

