# Supplementary material for: Development and Validation of the Single Item Narcissism Scale (SINS)
Source: PLoS One. 2014 Aug 5;9(8):e103469. doi: 10.1371/journal.pone.0103469 (PMC4122388; doi:10.1371/journal.pone.0103469)
Supplement: Appendix S1 — The Single-Item Narcissism Scale (SINS). This file includes the full text of the Single-Item Narcissism Scale (SINS). (DOCX) [file pone.0103469.s001.docx]

**Appendix S1 : The Single-Item Narcissism Scale (SINS)**

To what extent do you agree with this statement: "*I am a narcissist*." (Note: The word “narcissist” means egotistical, self-focused, and vain.)

1 2 3 4 5 6 7

not very very

true of me true of me
