# Supplementary material for: Development and Validation of the Single Item Narcissism Scale (SINS)
Source: PLoS One. 2014 Aug 5;9(8):e103469. doi: 10.1371/journal.pone.0103469 (PMC4122388; doi:10.1371/journal.pone.0103469)
Supplement: Appendix S2 — Study 11 experimental manipulation materials. This file includes the images that were used to manipulate ego threat versus ego boost in Study 11. (DOCX) [file pone.0103469.s002.docx]

**Appendix S2: Study 11 experimental manipulation materials.**

(i) Ego threat stimulus

***Security Check:****Enter****three****words shown in the image.*

**
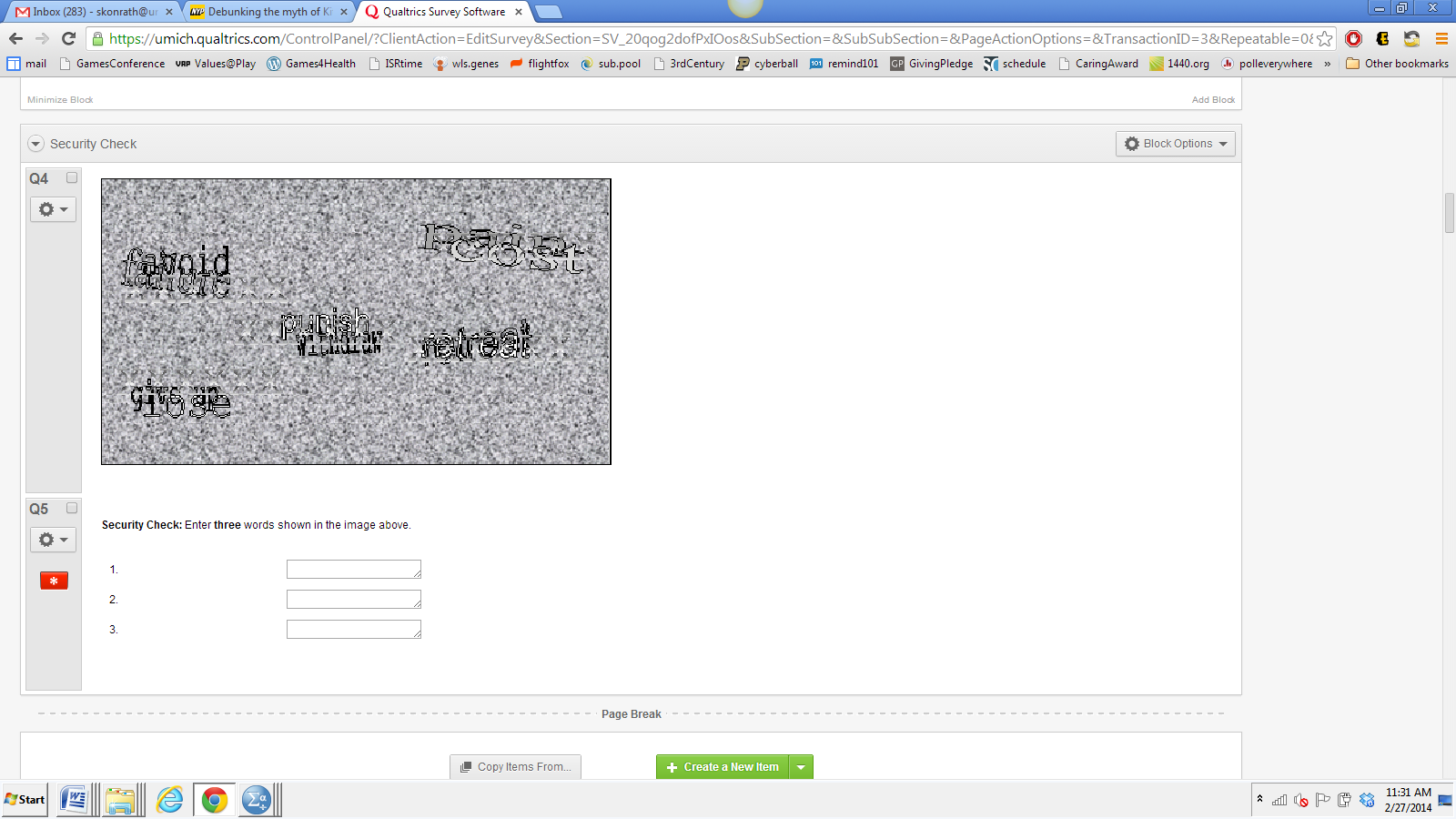
**

*Note: Words present include avoid, failure, give up, lose, punish, withdraw, pain, cost, retreat*

(ii) Ego boost stimulus

***Security Check:****Enter****three****words shown in the image.*


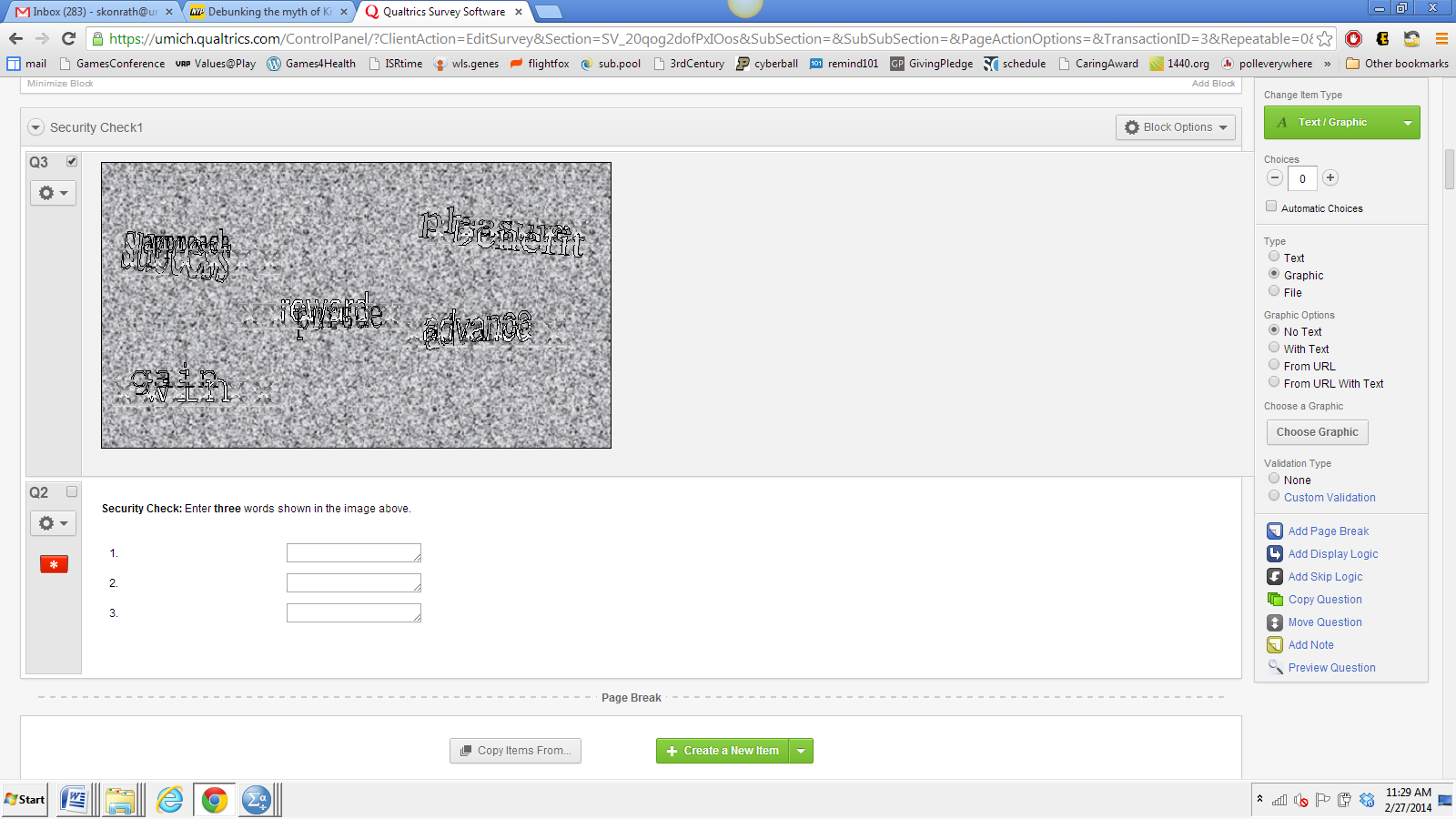


*Note: Words present include success, approach, gain, win, reward, pride, pleasure, benefit, advance*
